# Pollution and Weather Reports: Using Machine Learning for Combating Pollution in Big Cities

**DOI:** 10.3390/s21217329

**Published:** 2021-11-03

**Authors:** Cicerone Laurentiu Popa, Tiberiu Gabriel Dobrescu, Catalin-Ionut Silvestru, Alexandru-Cristian Firulescu, Constantin Adrian Popescu, Costel Emil Cotet

**Affiliations:** Robots and Production System Department, University Politehnica of Bucharest, Splaiul Independenței 313, 060041 Bucharest, Romania; tiberiu.dobrescu@upb.ro (T.G.D.); catalin.silvestru@upb.ro (C.-I.S.); alexandru.firulescu@stud.fiir.upb.ro (A.-C.F.); cpopescu1107@upb.ro (C.A.P.); costel.cotet@upb.ro (C.E.C.)

**Keywords:** pollution, sensors, machine learning, smart city

## Abstract

Air pollution has become the most important issue concerning human evolution in the last century, as the levels of toxic gases and particles present in the air create health problems and affect the ecosystems of the planet. Scientists and environmental organizations have been looking for new ways to combat and control the air pollution, developing new solutions as technologies evolves. In the last decade, devices able to observe and maintain pollution levels have become more accessible and less expensive, and with the appearance of the Internet of Things (IoT), new approaches for combating pollution were born. The focus of the research presented in this paper was predicting behaviours regarding the air quality index using machine learning. Data were collected from one of the six atmospheric stations set in relevant areas of Bucharest, Romania, to validate our model. Several algorithms were proposed to study the evolution of temperature depending on the level of pollution and on several pollution factors. In the end, the results generated by the algorithms are presented considering the types of pollutants for two distinct periods. Prediction errors were highlighted by the RMSE (Root Mean Square Error) for each of the three machine learning algorithms used.

## 1. Introduction

Pollution represents probably the most important topic in the last decade, an issue that is constantly discussed in the media, in government meetings and environment activities. For the last decades, air pollution has seemed to be impossible to manage and control, but this can be combated with the help from advancements in technology. By making use of the Internet of Things and machine learning (ML) technology, the pollution could be contained and controlled, as well as predict future rises in air contamination in urban areas [1].

Monitoring the air pollution levels implies the existence of a scale of the air quality, which can be measured with the help of sensor technology. The Air Quality Index (AQI) is based on the measuring of the liquid droplets and solid particles found in the air, which consists mostly of nitrogen dioxide (NO_2_), ozone (O3), sulphur dioxide (SO_2_) and carbon monoxide (CO). The key pollutants to calculate AQI are particulate matter (PM2.5 and PM10), which determines the class of air quality level (goof, satisfactory, moderately, poor, very poor and severe), with a range from 0 to 500; each category of AQI has an impact on human health and the environment. Each air pollutant has its own source and effects; thus, managing to get an image of the air pollution sources in an area based on the highest-level polluting particle, for example, a high level of nitrogen dioxide, tells us that in that area fossil fuel burning occurs, possible due to heavy traffic in the area, etc. [2].

AQI, also referred to as the Air Pollution Index (API) or Pollutant Standard Index (PSI), creates an image of the air quality in a specific range for every air pollutant. For example, PM2.5 is determined by fitting the arithmetic average of hourly values recorded in the last 24 h. The specific index is classified from 1 to 6, each index having a PM2.5 concentration measured in µg/m^3^. More details about the concentration of this particle are showed in Table 1.

Each index is represented by a colour and indicates a level of health concern, as well as the measures needed to be applied regarding the level of air contamination. The example from the table is the standard for the National Air Quality Monitoring Network of Romania [3].

In order to determine the exact value of AQI and to detect which air pollutants are responsible for this disaster, various sensors from several categories currently available could be used; for example, electrochemical sensors that are based on a chemical reaction between gases in the air and the electrode in a liquid inside a sensor, or photo ionization detectors, optical particle counters or even optical sensors [4].

By placing such sensors across urban areas, in combination with weather detection sensors and creating a connection algorithm between them, one can create live reports of AQI and determine potentially dangerous zones. With the help of ML, one also can create forecasts regarding air pollution and prevent further increase in the Air Quality Index. 

The paper is organized as follows: In Chapter 2, a literature review is presented. Chapter 3 includes details about the physical system. Chapter 4 presents details regarding the machine learning algorithm for combating pollution and the data collected and used in the case study. In Chapter 5 the results are analysed, while Chapter 6 discusses the conclusion of the research and future work.

## 2. Related Work

Awan et al. [5] used a long short-term memory recurrent neural network (LSTM RNN) to perform traffic flow forecasting, with time-series traffic flow, air pollution and atmospheric data collected from the open datasets. The goal was to find solutions to obtain an accurate prediction for road traffic forecasting. In their paper, Zhu et al. [6] proposed refined models to predict the hourly air pollution concentration on the basis of meteorological data of previous days by formulating the prediction over 24 h as a multi-task learning (MTL) problem. Kalajdjieski et al. [7] evaluated four different architectures that utilize camera images to estimate the air pollution in big cities. Accurate air pollution prediction could be obtained by combining sensor data with camera images. Castelli et al. [8] demonstrated that SVR (Support Vector Regression) with an RBF (radial basis function) kernel could assure accurate predictions of hourly pollutant concentrations, such as PM2.5, NO_2_, SO_2_ and carbon monoxide. Delavar et al. [9] presented a comparative study of NARX (Nonlinear Autoregressive Exogenous Model), ANN (Artificial Neural Networks), GWR (Geographically Weighted Regression) and SVR machine learning methods in order to predict air pollution. The results revealed that the NARX method was the optimum one for their case study.

Wang et al. [10] investigated the boundaries of Land-Use Regression (LUR) approaches and the potential of two different machine learning models: Artificial Neural Networks and Gradient Boost. They conclude that for the same pollutants, machine learning exhibited superior performance over LUR, demonstrating that LUR performance could benefit from understanding how the explanatory variables were expressed in the machine learning models. Guan and Sinnott [11] used ANN models and LSTMs to predict high PM2.5. Their results show that accurate prediction was obtained with LSTM.

Adityia et al. [12] used logistic regression to detect whether a data sample is either polluted or not polluted. The authors considered to predict future values of PM2.5 based on the previous PM2.5 readings. In [13], several machine learning methods were analysed to predict the ozone level (O_3_) in the Region of Murcia, Spain. The authors excluded from their study SO_2_, NO_x_(Nitrogen Oxides), NH_3_ (Ammonia) and CO.

In [14], a machine learning model that combines sparse fixed station data with dense mobile sensor data was used to estimate the air pollution in Sydney. Shaban et al. [15] presented three machine learning algorithms to build accurate forecasting models for one-step and multi-step ahead of concentrations of ground-level ozone, nitrogen dioxide and sulphur dioxide.

Zang and Woo [16] show that the hybrid distributed, fixed IoT sensor system is effective in predicting air quality. In [17], the authors are presenting a review on studies related to air pollution prediction using machine learning algorithms based on sensor data in the context of smart cities. 

Lim et al. [18] show that data collected from mobile sampling with multiple low-cost sensors could be used to model and map street-level air pollution levels in urban locations. Kang et al. [19] reviewed the published research results relating to air quality evaluation using methods of artificial intelligence, decision trees and deep learning.

In [20] is proposed a methodology to evaluate and compare deep learning models for multivariate time series forecasting, which includes lagged transformations, hyper-parameter tuning, statistical tests and multi-criteria decision making. In [21] are presented applications of deep learning (DL) techniques to predict air pollution time series. In their case study, 8 h-averaged surface ozone concentrations were predicted using deep learning consisting of a recurrent neural network (RNN) with long short-term memory 

Song et al. [22] proposed a machine learning framework (Deep-MAPS) to for fine-granular PM2.5 inference based on fixed and mobile air quality sensing data. Ameer et al. [23] performed pollution prediction using four advanced regression techniques and present a comparative study to determine the best model for accurately predicting air quality with reference to data size and processing time.

In [24] is presented a comparative study of various statistical and deep learning methods to forecast long-term pollution trends for PM2.5 and PM10. The case study is based on data from sensors available in a big city from India. In their paper, Chen et al. [25] compared several Aerosol Optical Depth-PM2.5 models, including Extra Trees (ET), Random Forest (RF), Deep Neural Network (DNN), and Gradient Boosting Regression Tree (GBRT). Their results indicate that the ET model performs best in terms of the model effectiveness and feature interpretation on the training dataset. Lana et al. [26] presented a methodology based on the construction of regression models to predict levels of different pollutants (CO, NO, NO_2_, O_3_ and PM10) based on traffic data and meteorological conditions, from which an estimation of the predictive relevance (importance) of each utilized feature can be estimated by virtue of their particular training procedure. The study was done considering historic traffic and pollution data of the city of Madrid, Spain.

Liang et al. [27] conducted a study using zero-inflated negative binomial models to estimate the association between long-term county-level exposures to NO_2_, PM2.5 and O_3_ and county-level COVID-19 case-fatality and mortality rates in the United States. In [28], the authors calculated the wildfire-smoke-related health burden and costs in Australia for the most recent 20 fire seasons. Their results show that the 2019–2020 season was a major anomaly in the recent record, with many smoke-related premature deaths in addition to a large number of hospital admissions for cardiovascular and respiratory disorders.

## 3. The Physical System

**Figure 1:**
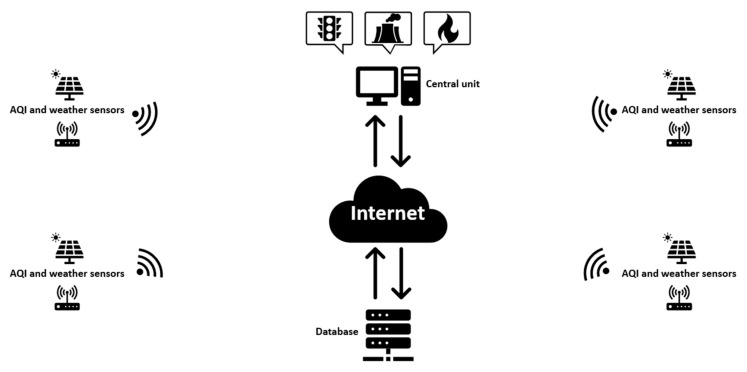
IoT block diagram of the physical system.

The base system presented in the paper is an IoT system block composed of air pollutant and weather sensors, a database server and a central unit that will implement the ML algorithm and control other systems for maintaining the levels of AQI in good parameters. All these components will be permanently connected via cloud technology. The diagram of the system is presented in Figure 1 [29], and each component will be detailed in the next subsections.

### 3.1. AQI Sensors

The main workers of this system are the AQI sensors, which detects the levels of air pollutants based on their specifications. A complete report of the air quality implies the utilization of various sensors simultaneously, each one dedicated to one or more of the air pollutants. 

The main kind of sensor used is the Optical Particulate Counter, which is used for measuring the PM. The optical sensors can identify particle pollution with sizes of 10 and 2.5 micrometres or even smaller. Since PM2.5 and PM10 consist of solid particles and liquid droplets, these optical sensors are the best fit in detecting the numbers of such particles in the air. Conversion from particle counts to PM mass is based on a theoretical model. The measured signal depends on a variety of parameters, such as particle shape, colour, density, humidity, refractive index and so on. [4] 

Other optical sensors can be used for the detection of carbon monoxide and carbon dioxide by measuring the absorption of infrared light. These sensors can be found mostly in indoor smoke detectors, as they can alarm in the case of fire. A disadvantage for these kinds of sensors is the fact that they require periodic calibration and the cleaning of their optical unit.

Another primary element in air quality monitoring is the gas sensor. These sensors come in a variety of working principles, each one sensitive to a specific gas and a specific concentration. Let us take for the first example a gas sensors with semiconductors. This type of gas sensor can use a metal oxide semiconductor or a polymer semiconductor, both functioning in the same manner: the conductivity of the sensing element will vary when exposed to the gas to be measured, also indicating the gas concentration. Another kind of gas sensor is the catalytic one, which reacts with the gases, creating a variation in the heat energy. Another example of a gas sensor is the electrochemical one, in which a chemical reaction takes place between two electrodes made from catalytic metals. These sensors can be further classified in two types: potentiometric and amperometric [30].

The gas sensors, even if they offer a good sensitivity (from mg/m^3^ to µg/m^3^) and a fast response time, are susceptible to external factors, such as temperature and humidity variations [4]. For this reason, the use of these sensors without some data regarding the meteorological changes can affect the results of the measured gasses.

### 3.2. Weather Sensors

The use of air pollutant sensors independently cannot offer an AQI good enough to create a report of the pollution origin. Air currents, temperature, humidity and atmospheric pressure play an important role in the detection of pollution and the determination of AQI. Thus, the use of sensors capable of detecting these variables in accordance with the sensors mentioned in the previous subsection can offer the most data to create a live report of the AQI, as well as creating a forecast of the weather and AQI in the next days. These weather sensors also have the important role of calibrating the data of the gas sensors, since they can be affected by the changes in meteorology.

### 3.3. Data Transmission

The data collected by the sensors must be collected and stored in a database. This operation must be done in a fast-paced manner, in a way that the data cannot be altered or lost during the transmission. To collect the data from the sensors placed across the urban area one can use multiple ways of communication. First, we must decide which type of connection can be used for the sensor: node-wired or wireless. In the case of a wired connection, an ethernet or an optic fibre can make for a solid route of data transmission, the only impediment being the necessity of pulling long cables and connecting them to the system. If the space and architecture of the place where the sensor node is installed cannot offer the possibility of implementing such a wired connection, we can call for a wireless one. A wireless connection can be done mainly via Wi-Fi, which will require the presence of a router connected to the internet in the proximity of the sensor node, or the installation of a GSM (Global System for Mobiles) module with the sensor node, thus allowing the use of GSM antennas in the vicinity. The last option is probably the most suited one in urban areas since GSM antennas can be present everywhere [31,32].

### 3.4. The Database and the Central Unit

The last components of the system consist of a database and a central processing unit, both being interconnected and in permanent communication with the sensor nodes.

The database has the role of collecting the data offered by the sensor nodes and creating reports of the AQI and weather status. By maintaining the data in an organized form, one can see the evolution of the AQI and create algorithms that can offer us a better understanding of the air pollution status and forecasts to prevent possible natural disasters or health warnings. Thus, we need to implement a mathematical system that process al these data.

Implementing a central processing unit with the use of machine learning technology can offer us all the information we need to combat air pollution. This unit can read the reports collected in the database and compare the data, creating a forecast of the AQI and offering information to find possible sources of pollution based on the data, such as the possibility of a fire, or to alert high levels of harmful particle in the air coming from a factory, or to control traffic lights in order to drain congested traffic. Such a system is presented in the next section of this paper.

## 4. Machine Learning in Combating Pollution

In this paper, machine learning is used to predict behaviours regarding the air quality index near the six air quality sensing units installed in Bucharest by the Romanian National Environmental Protection Agency. The predicted behaviours should be used as an incentive to act, make the required changes in cleaning the air and recommending the population to avoid the area.

### 4.1. Data Collection and Their Use in Different Machine Learning Algorithms

The first step in using the ML algorithms is preparing the data; for this purpose, the data were split into 70% training data and 30% test data. Our data consist of 8700 entries from March 2019 to February 2020, during the height of the COVID-19 pandemic, when open circulation was not permitted, and another 8700 entries from March 2020 to February 2021, a period in which, depending on the country, restrictions were lifted. The data were separated into five columns: Temperature in Celsius, CO quantity, NO_2_ quantity, SO_2_ quantity and PM2.5 quantity. Afterwards, a suitable algorithm needs to be selected; some have tried using artificial neural networks [1,29,33,34], but others have used machine learning algorithms [1,13,29].

The most common machine learning algorithm used in other papers is Random Forests; this algorithm works by generating multiple decision trees while it is being trained. A decision tree splits data into separate categories finishing only when there are no more distinctive elements that can be further classified. Each decision tree becomes a class on its own and each tree should be uncorrelated to another [35].

By being a core component of the Random Forests approach, decision-tree learning is natural to be used on its own. This approach uses a singular decision tree to map the data, make observations and predict outcomes. 

Another used algorithm is Support Vector Machines, which can do both classification and regression tasks. SVMs split the data along a hyperplane, a boundary that helps to classify data. The support vectors are the nearest points of each class, equidistant from the hyperplane. Any modification to these support vectors changes the classification [36].

One of the aims of smart cities is to act based on data obtained through sensors. However, it must be noted that the sensors may cause failures and errors when obtaining data; hence, it is necessary to develop a model to predict the values of interest in order to control air quality [13].

The data used for the case study were obtained from one of the six atmospheric stations in Bucharest. Each station is placed in key points of the city, where the number of people commuting and living in the area is high. The data were collected every hour, covering the climatic parameters and chemical elements present in the air. The stations can be different regarding the equipment, but they offer valuable data.

### 4.2. Examples of Using the Results Obtained after Processing with Machine Learning 

The data collected and processed via the algorithms presented earlier can be used to create a forecast of the weather and the AQI. Since we live in the era of technology and every device is connected to the internet, no matter its dimensions or purpose, IoT technology became the main picture in the eyes of developers, companies and scientists. By connecting the system presented in this paper to the cameras inspecting the traffic and the big sources of air pollution, such as factories and landfills, one can create a complex system that can warn against and combat air pollution. 

**Figure 2:**
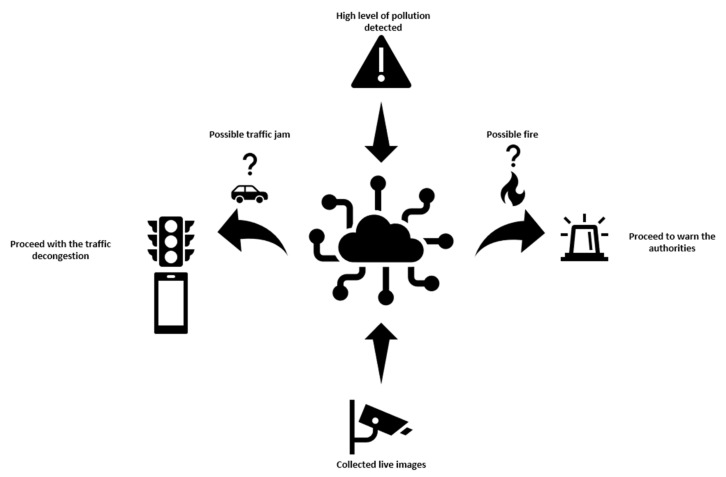
Graphic presentation of the complex system at work.

For example, if near an atmospheric station is recorded an increase in the NO_2_ particles, one can conclude that these particles are originated from the burning of fossil fuels, whose main source is from the use of automobiles. From these data one can suppose that a traffic jam takes place near the station. The system can further check via the traffic cameras that this can really be the source of the air pollutant. If yes, the system can start searching for a way of manipulating the traffic lights so that the circulation can be improved, eventually warning other traffic participants to take other routes. The principle diagram of such a system is presented in Figure 2.

Another example would be when high levels of CO_2_ are detected. This can further indicate a potential fire somewhere near the station. The source of the particles could be everywhere in a fairly wide radius. To limit the space, wind sensors can help find the source. By tracking the wind speed and direction, the system can create a map of possible sources for the pollutant. It can then search for the source visually with the help of the cameras, eventually warning the authorities of a potentially detected fire.

### 4.3. Using Machine Learning for Data Collected from an Atmospheric Station in Bucharest, Romania

**Figure 3:**
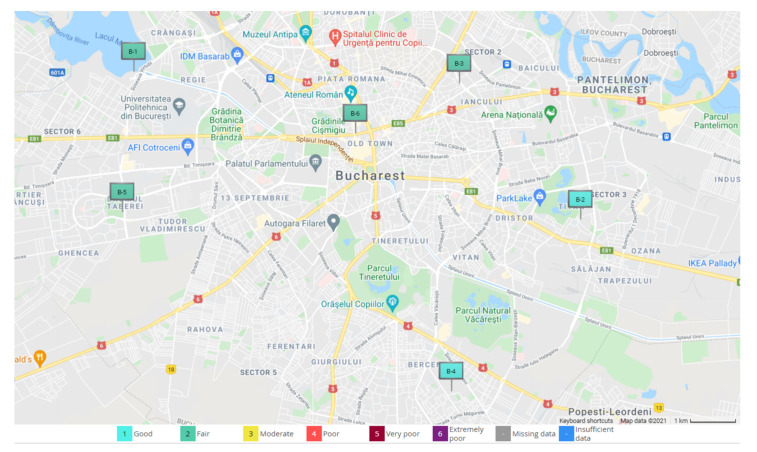
Emplacement of the B-1 to B-6 atmospheric stations in Bucharest [3].

**Figure 4:**
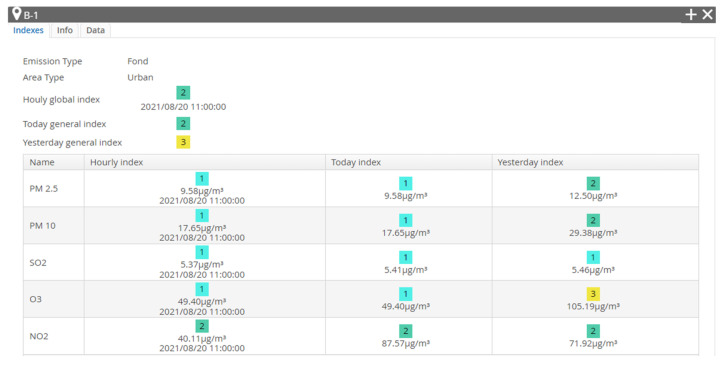
Indexes collected by station B-1 [3].

**Figure 5:**
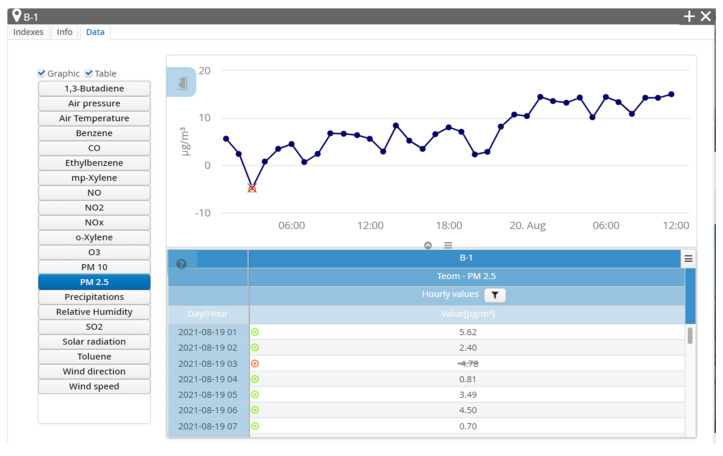
Data collected by station B-1 regarding the PM 2.5 pollutant [3].

As further presented in this paper, data were collected from one of the six atmospheric stations in Bucharest set in relevant areas of Bucharest. These are marked as B-x, where x is the number of the station from 1 to 6. Their emplacement is shown in Figure 3. These stations are equipped with a multitude of sensors—gas, optical and meteorological ones—and transmit the information collected to the database, creating reports for every air pollutant. Such reports are showed in Figure 4 and Figure 5, where visible are the graphics and values collected by the B-1 station, placed in the vicinity of Lake Morii, the station from which the data used in this article were collected as input data for processing with specific machine learning algorithms.

Figure 3 shows the map with the positioning of the six atmospheric stations in Bucharest, from which data were taken about the level of pollution in that area, as we mentioned that these stations are positioned in crowded areas of Bucharest. In this figure, too, at the bottom of it, one can notice the pollution levels highlighted by distinct colours, presented in Table 1, for all cases that may exist.

The acquired data on the pollution level are diverse (Figure 2) and can reflect in time the evolution of the concentration of pollutants existing in the monitored area, thus providing access, through the pollution monitoring platform of Bucharest, to these data in real time but also to the archive of data stored over the past years. A concrete example in this sense is presented in Figure 5, where the evolution of PM2.5 in a certain time interval is highlighted. 

In this paper, through machine learning techniques, we highlighted the RMSE (Root Mean Square Error) value of the different models that predict the evolution of temperature. RMSE is the standard deviation of residues (prediction errors).

Therefore, we analysed based on several algorithms the evolution of temperature depending on the level of pollution, referring to several pollution factors.

These tests were initially performed for the past years, specifically to be able to validate the model of the proposed algorithm to be implemented. In this paper are presented the conclusive results from the proposed algorithms in relation to the types of pollutants, for two distinct time periods, with the aspects clarified in the next chapter.

In order to eloquently synthesize the work stages, but also the specific implications necessary to be realized for carrying out this research, we present a descriptive diagram with all the steps taken to carry out this research.

**Figure 6:**
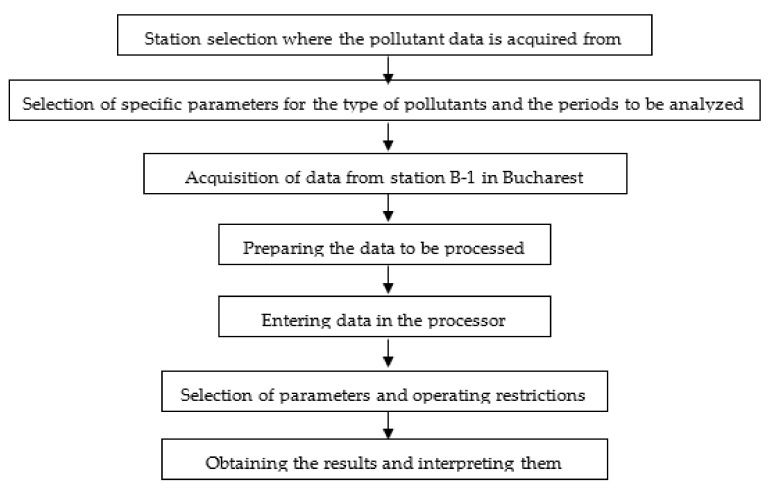
The information flow used to obtain results using machine learning.

Figure 6 shows the basic information flow that led to the results:

## 5. Results and Discussion

The study was conducted for the period March 2019–February 2021. For the analysis, three algorithms were used, and we wanted to highlight the evolution of temperature according to the different pollutants (nitrogen dioxide, sulphur dioxide, carbon monoxide and powder in suspension).

The algorithms used for the analysis are the linear regression algorithm, support vector machines with Gaussian kernel and Gaussian process regression (GPR) using an exponential kernel.

Gaussian process regression models are nonparametric kernel-based probabilistic models.

Consider the training set {(x_i_,y_i_); i = 1,2,...,n}, where x_i_∈ℝd and y_i_∈ℝ, drawn from an unknown distribution. A GPR model addresses the question of predicting the value of a response variable ynew, given the new input vector xnew, and the training data. A linear regression model is of the form

y = x^T^β + ε,

where ε∼N(0, σ^2^). 

The error variance σ^2^ and the coefficients β are estimated from the data. A GPR model explains the response by introducing latent variables, f(x_i_), i = 1, 2, ..., n, from a Gaussian process (GP), and explicit basis functions, h. The covariance function of the latent variables captures the smoothness of the response and basic functions project the inputs x into a p-dimensional feature space.

A GP is a set of random variables, such that any finite number of them have a joint Gaussian distribution. If {f(x), x ∈ ℝ^d^} is a GP, then given n observations x_1_, x_2_, ..., x_n_, the joint distribution of the random variables f(x_1_), f(x_2_), ..., f(x_n_) is Gaussian. A GP is defined by its mean function m(x) and covariance function, k(x, x′); that is, if {f(x), x ∈ ℝ^d^} is a Gaussian process, then E(f(x)) = m(x) and Cov[f(x), f(x′)] = E[{f(x) − m(x)} {f(x′) − m(x′)}] = k(x, x′)” [37].

For the period March 2019–February 2020, the pollution sensors from the station for which the case study was performed did not provide enough data and thus could not perform a complete analysis, but the data were processed, and the graphs obtained presented in comparison with charts for the period March 2020–February 2021.

**Figure 7:**
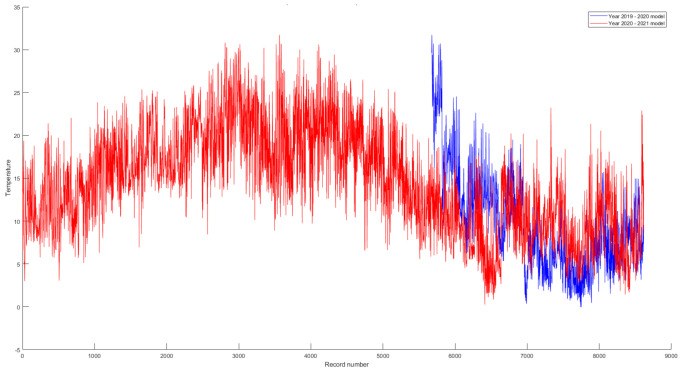
Comparison between the optimised GPR models.

To highlight the difference between the two periods, namely, March 2019–February 2020 and March 2020–February 2021, the graphs presented in Figure 7 were drawn.

Both models are of the GPR type with an exponential kernel and were optimized. The introduction of the model without optimization was considered redundant since its performance was lower than the optimized one. On the red chart (March 2020–February 2021), around the records 2200–4400 are the summer months in which there is a natural increase in temperature, but in the rest of the chart one can see a tendency to increase temperatures over time.

The linear regression models were trained in 7 s while using a linear method. The SVM models were trained using a fine Gaussian function with the kernel scale of 0.5 and was trained in 11 s. The GPR models were optimised using a Bayesian optimization method with the acquisition function: expected improvement over an hour training time, the best kernel function found was a non-isotropic exponential.

Having a very large volume of input data and considering that it is not relevant to be presented in full in the article, we have highlighted in the Table 2, some such data in order to exemplify the type and form of this input data used within machine learning algorithms.

The key inputs were: Temperature (in Celsius), NO_2_ concentration, SO_2_ concentration, CO concentration and PM2.5 concentration. All records in the dataset period were considered while training the models, and no filters were considered to be needed. Rows where one or more entries were missing have been used in each individual pollutant model and were shown but not considered in the model regarding all pollutants.

The output was a model that is used to predict how the temperature will fluctuate during the year.

Figure 8, Figure 9, Figure 10, Figure 11, Figure 12, Figure 13, Figure 14, Figure 15, Figure 16, Figure 17, Figure 18, Figure 19, Figure 20, Figure 21 and Figure 22 show the graphs obtained based on the three algorithms: linear regression algorithm, support vector machines with the Gaussian kernel and Gaussian process regression, compared for the periods March 2019–February 2020 and March 2020–February 2021, with specifying that the data for the period March 2019–February 2020 were not complete.

Each model has a figure considering all the pollutants together as well as a figure of each one separately (only SO_2_, only NO_2_, only CO and only PM2.5), given that all the pollutants together give us a different model overall than when seeing the influence of only one pollutant.

In each analysis, the model and all the data were presented without the separation of the test and training data sets.

Each model has a figure considering all pollutants, only SO_2_, only NO_2_, only CO and only PM2.5; this considering all pollutants gives us a different model overall, compared to seeing the influence of only one pollutant.

GPR models were optimized in MATLAB using the Bayesian Optimization Algorithm using functions such as expected improvement. The Bayesian optimization algorithm tries to minimize the function of the model in a limited field, and the family of improvement functions evaluates the values that bring an improvement within the model and ignores those that do not minimize the model. The expected improvement functions use the following relation [38]:

EI(x, Q) = E_Q_[max(0, μ_Q_(x_best_) − f(x))]

where x_best_ as the location of the lowest posterior mean.

In Figure 18a one can see (in yellow) the identified model that makes a prediction of the temperature rise considering all types of pollutants, but due to a lack of data, cannot identify a correct prediction.

The model in Figure 13a is unoptimized, and due to the Gaussian kernel, it looks like the model in Figure 18a (discussed above), the lack of data not being able to lead to a correct prediction.

**Figure 8:**
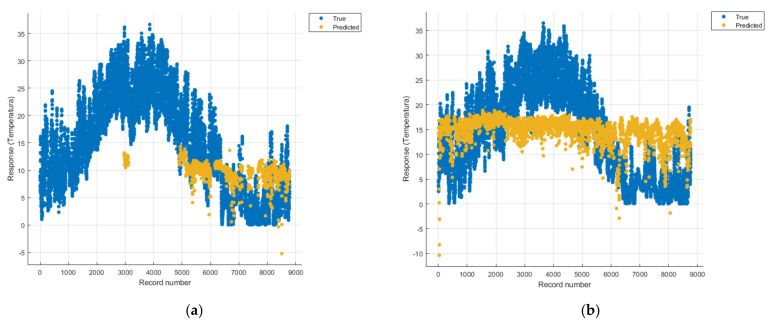
Prediction considering all pollutants—Linear Regression. (**a**) March 2019—February 2020, (**b**) March 2020—February 2021.

The linear regression model in Figure 8a is unsuitable for analysis because no linear function can be identified to model the data correctly.

Considering the pollutants individually, the linear regression graphs (Figure 9, Figure 11, Figure 12 and Figure 13) offer a more detailed understanding than the other graphs. The GPR graphs (Figure 19, Figure 20, Figure 21 and Figure 22) validate the results obtained with linear regression and, following the analysis of the SVM model (Figure 14, Figure 15, Figure 16 and Figure 18), no conclusions can be drawn.

For the period March 2020–February 2021, the graphs were drawn based on complete data sets, the predictions obtained being much more suggestive. 

**Figure 9:**
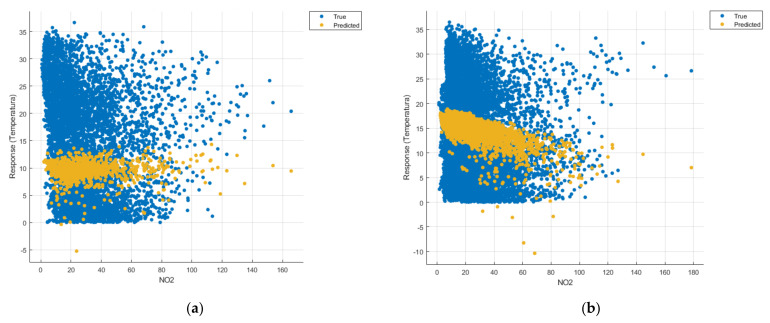
Prediction considering only NO_2_—Linear Regression. (**a**) March 2019—February 2020, (**b**) March 2020—February 2021.

**Figure 10:**
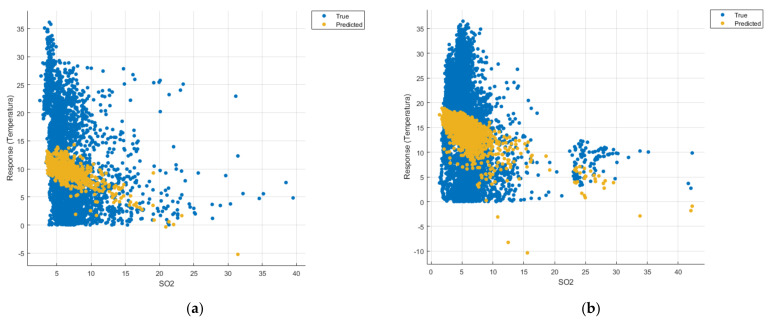
Prediction considering only SO_2_—Linear Regression. (**a**) March 2019—February 2020, (**b**) March 2020—February 2021.

In the graphs in Figure 9b, Figure 14b and Figure 19b, where the influence of the amount of nitrogen dioxide on the temperature was considered, a tendency to increase the temperature at higher NO_2_ concentrations can be observed. In contrast, in the graphs in Figure 10b, Figure 15b and Figure 20b, where the influence of the amount of sulphur dioxide on the temperature was considered, a tendency to decrease the temperature at higher SO_2_ concentrations can be observed.

**Figure 11:**
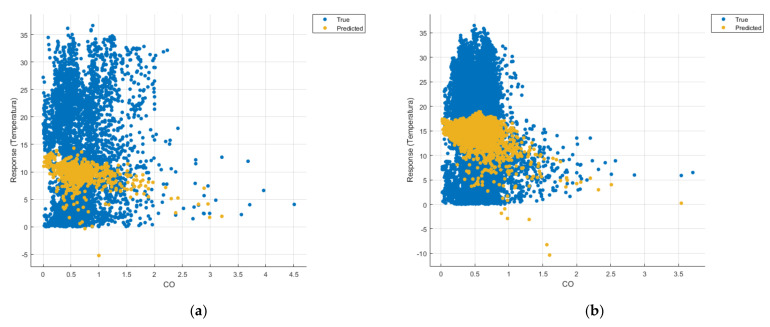
Prediction considering only CO—Linear Regression. (**a**) March 2019—February 2020, (**b**) March 2020—February 2021.

Similarly, in the graphs in Figure 11b, Figure 16b and Figure 21b, where the influence of monoxide amounts on temperature was influenced, a tendency to decrease the temperature at higher CO concentrations can be observed.

**Figure 12:**
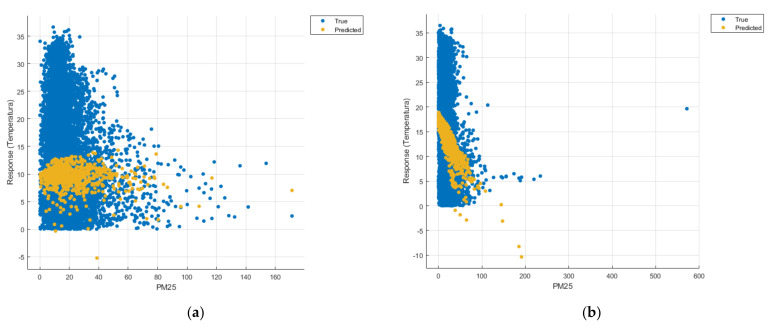
Prediction considering only PM2.5—Linear Regression. (**a**) March 2019—February 2020, (**b**) March 2020—February 2021.

**Figure 13:**
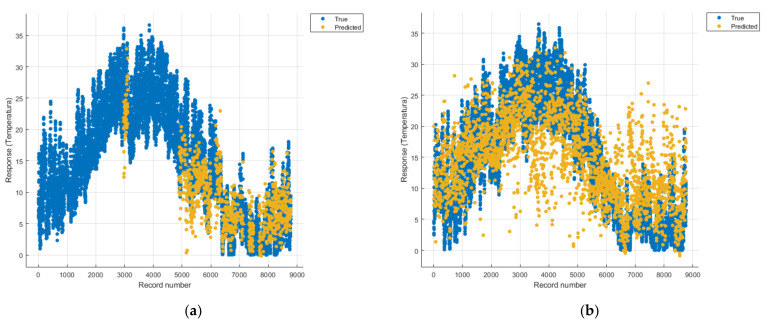
Prediction considering all pollutants—Fine Gaussian SVM. (**a**) March 2019—February 2020, (**b**) March 2020—February 2021.

**Figure 14:**
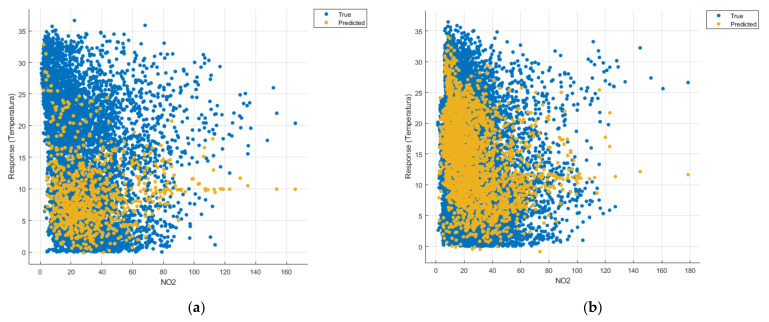
Prediction considering only NO_2_—Fine Gaussian SVM. (**a**) March 2019—February 2020, (**b**) March 2020—February 2021.

**Figure 15:**
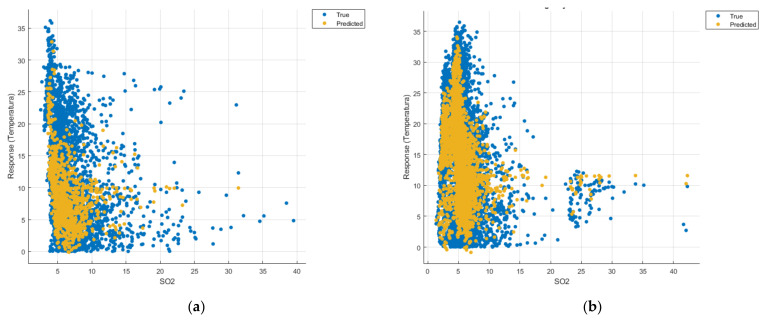
Prediction considering only SO_2_—Fine Gaussian SVM. (**a**) March 2019—February 2020, (**b**) March 2020—February 2021.

**Figure 16:**
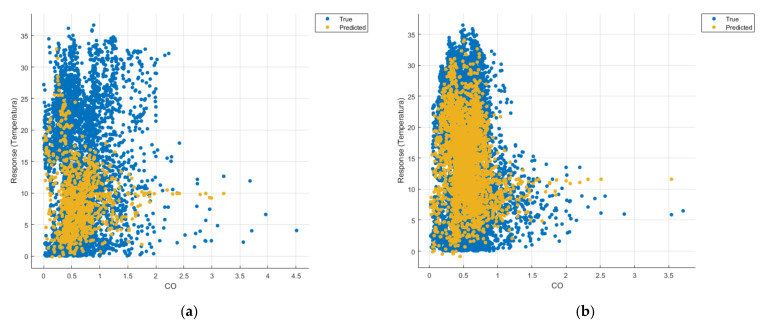
Prediction considering only CO_2_—Fine Gaussian SVM. (**a**) March 2019—February 2020, (**b**) March 2020—February 2021.

**Figure 17:**
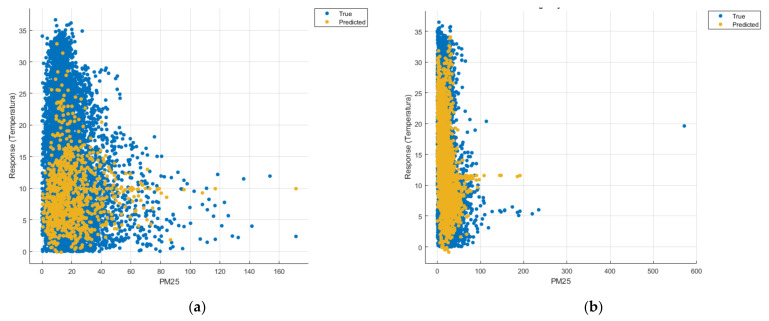
Prediction considering only PM2.5—Fine Gaussian SVM (**a**) March 2019—February 2020, (**b**) March 2020—February 2021.

**Figure 18:**
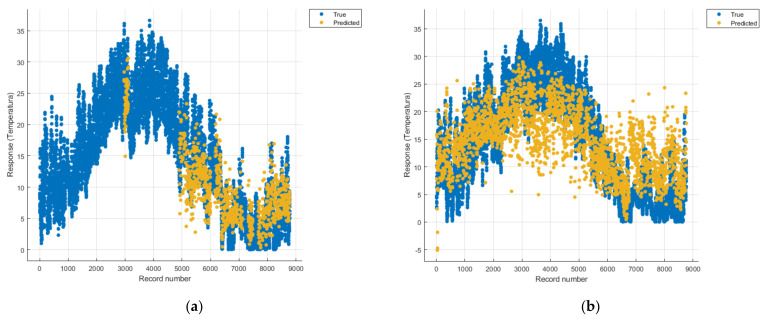
Prediction considering all pollutants—optimised GPR. (**a**) March 2019—February 2020, (**b**) March 2020—February 2021.

**Figure 19:**
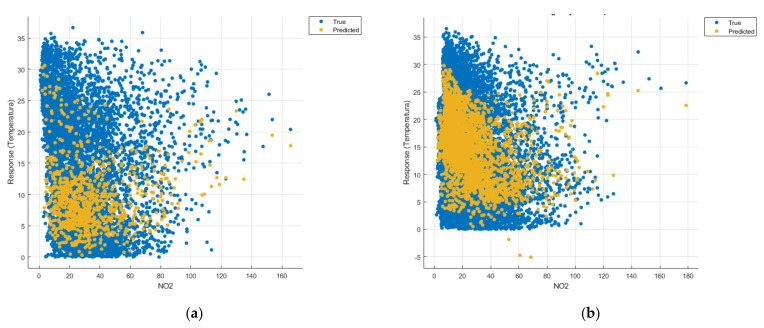
Prediction considering only NO_2_—optimised GPR. (**a**) March 2019—February 2020, (**b**) March 2020—February 2021.

**Figure 20:**
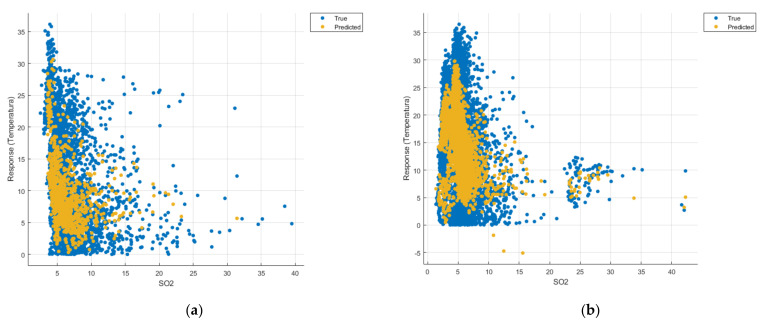
Prediction considering only SO_2_—optimised GPR. (**a**) March 2019—February 2020, (**b**) March 2020—February 2021.

**Figure 21:**
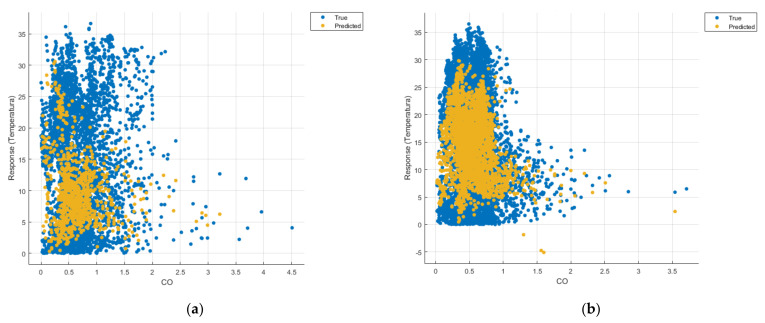
Prediction considering only CO—optimised GPR. (**a**) March 2019—February 2020, (**b**) March 2020—February 2021.

**Figure 22:**
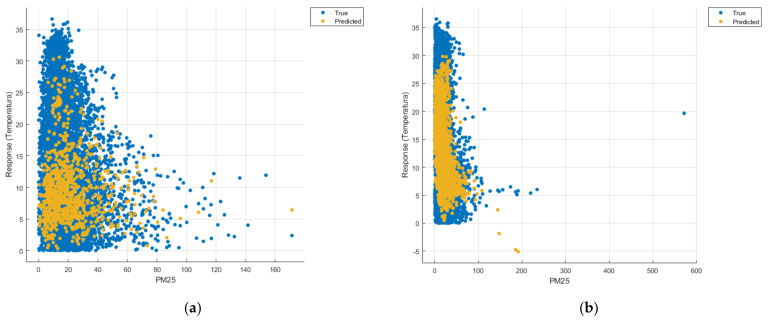
Prediction considering only PM2.5—optimised GPR. (**a**) March 2019—February 2020, (**b**) March 2020—February 2021.

In the case of the graphs in Figure 12b, Figure 17b and Figure 22b, where the influence of the amount of PM2.5 particles on temperature was considered, no significant increase in temperature was observed.

To help decide the performance of the models, we used the Root Mean Square Error (RMSE) value (Table 3). RMSE is the standard deviation of the residuals (prediction errors). Residuals are a measure of how far from the regression line data points are. The lower the RMSE value the better the performance.

It was noted that the worst algorithm for the used datasets was the Linear Regression, and the Gaussian kernel SVM was the second worst, fitting the data a bit better; however, the best result was obtained by using an optimized GPR algorithm, with which much smaller error is obtained compared to the other methods.

## 6. Conclusions

The influence of atmospheric pollutants on temperature is significant, and these pollutants must be constantly monitored to make predictions on temperature depending on their quantity. Pollution sensor systems used for data acquisition are currently used in all major cities around the world to monitor the degree of air pollution. Implementing a machine-learning algorithm to process the data purchased from these sensors can provide solutions to maintain the optimal parameters for the global temperature, which has constantly been increasing in the last decades.

In the research described here, the emphasis was on using different machine learning algorithms to predict the evolution of temperature in a crowded area of Bucharest, Romania. Various algorithms were selected to model the impact of several pollution factors on the level of the temperature. In order to validate the model, data were collected from one of the six atmospheric stations set in relevant areas of Bucharest. The use of sensors for data acquisition is essential for such a system that allows processing a substantial volume of data to make predictions. This aspect was highlighted by comparing the graphs obtained based on data from the two time periods, namely, March 2019–February 2020 and March 2020–February 2021. It was noted that if the data were insufficient for technical reasons that the authors could not solve (March 2019–February 2020), some complete results were not obtained, even if the same algorithm was used for the data processing. 

The proposed algorithms considering the types of pollutants for two distinct periods generated some relevant results. Through machine learning techniques, the RMSE was highlighted for values of different models that predict temperature evolution. The models that led to the most complete and accurate results were Gaussian process regression (GPR), used with the Bayesian Optimization Algorithm. 

Further research will combine the present results with camera images to analyse and predict air pollution in various big cities. Future research plans also include developing a platform to offer solutions for traffic recommendations based on air pollution predictions.

## Figures and Tables

**Table 1:** Specific index for the concentration of PM2.5.

Field of Particulate Matter PM2.5 Concentration (µg/m^3^)	Specific Index
0–10	1
10–20	2
20–25	3
25–50	4
50–75	5
75–800	6

**Table 2:** Examples of input data used in machine learning.

B1-Atmospheric Station				
NO_2_ (µg/m^3^)	SO_2_ (µg/m^3^)	CO (µg/m^3^)	PM2.5 (µg/m^3^)	Temperature (°C)
23.48	4.84	0.75	23.57	4.70
17.72	4.80	0.71	21.38	4.37
24.09	4.63	0.80	18.42	3.70
29.23	4.70	0.91	21.79	2.77
30.37	4.61	0.94	26.19	2.51
36.45	4.90	0.97	24.84	2.56
57.11	5.29	1.19	24.47	2.74
50.03	5.21	1.13	25.29	3.41

**Table 3:** Specific index for the concentration of PM2.5.

	Model RMSE	
	2019–2020 Model	2020–2021 Model
Linear Regression	7.035	8.659
SVM	5.213	7.73
Optimised GPR	4.753	6.9

## Data Availability

Not applicable.
